# Supplementary material for: A field guide for the compositional analysis of any-omics data
Source: Gigascience. 2019 Sep 23;8(9):giz107. doi: 10.1093/gigascience/giz107 (PMC6755255; doi:10.1093/gigascience/giz107)
Supplement: giz107_GIGA-D-19-00052_Original_Submission [file giz107_giga-d-19-00052_original_submission.pdf]

GigaScience

A field guide for the compositional analysis of any-omics data

--Manuscript Draft--

**Manuscript Number:**	GIGA-D-19-00052
**Full Title:**	A field guide for the compositional analysis of any-omics data
**Article Type:**	Technical Note
**Funding Information:**	
**Abstract:**	Next-generation sequencing (NGS) has made it possible to determine the sequence and relative abundance of all nucleotides in a biological or environmental sample. Today, NGS is routinely used to understand many important topics in biology from human disease to microorganism diversity. A cornerstone of NGS is the quantification of RNA or DNA presence as counts. However, these counts are not counts per se: the magnitude of the counts are determined arbitrarily by the sequencing depth, not by the input material. Consequently, counts must undergo normalization prior to use. Conventional normalization methods require a set of assumptions: they assume that the majority of features are unchanged, and that all environments under study have the same carrying capacity for nucleotide synthesis. These assumptions are often untestable and may not hold when comparing heterogeneous samples (e.g., samples collected across distinct cancers or tissues). Instead, methods developed within the field of compositional data analysis offer a general solution that is assumption-free and valid for all data. In this manuscript, we synthesize the extant literature to provide a concise guide on how to apply compositional data analysis to NGS count data. In doing so, we review zero replacement, differential abundance analysis, and within-group and between-group coordination analysis. We then discuss how this pipeline can accommodate complex study design, facilitate the analysis of vertically and horizontally integrated data, including multi-omics data, and further extend to single-cell sequencing data. In highlighting the limitations of total library size, effective library size, and spike-in normalizations, we propose the log-ratio transformation as a general solution to answer the question, "Relative to some important activity of the cell, what is changing?". Taken together, this manuscript establishes the first fully comprehensive analysis protocol that is suitable for any and all -omics data.
**Corresponding Author:**	Thomas P Quinn
**Corresponding Author Secondary Information:**	
**Corresponding Author's Institution:**	
**Corresponding Author's Secondary Institution:**	
**First Author:**	Ionas Erb
**First Author Secondary Information:**	
**Order of Authors:**	Ionas Erb
	Greg Gloor
	Cedric Notredame
	Mark F. Richardson
	Tamsyn M. Crowley
	Thomas P Quinn
**Order of Authors Secondary Information:**	
**Additional Information:**	
**Question**	**Response**

Powered by Editorial Manager® and ProduXion Manager® from Aries Systems Corporation

Are you submitting this manuscript to a special series or article collection?	No
**Experimental design and statistics**<br><br>Full details of the experimental design and statistical methods used should be given in the Methods section, as detailed in our Minimum Standards Reporting Checklist. Information essential to interpreting the data presented should be made available in the figure legends.<br><br>Have you included all the information requested in your manuscript?	Yes
**Resources**<br><br>A description of all resources used, including antibodies, cell lines, animals and software tools, with enough information to allow them to be uniquely identified, should be included in the Methods section. Authors are strongly encouraged to cite Research Resource Identifiers (RRIDs) for antibodies, model organisms and tools, where possible.<br><br>Have you included the information requested as detailed in our Minimum Standards Reporting Checklist?	Yes
**Availability of data and materials**<br><br>All datasets and code on which the conclusions of the paper rely must be either included in your submission or deposited in publicly available repositories (where available and ethically appropriate), referencing such data using a unique identifier in the references and in the "Availability of Data and Materials" section of your manuscript.<br><br>Have you have met the above requirement as detailed in our Minimum	Yes

Standards Reporting Checklist?	

Powered by Editorial Manager® and ProduXion Manager® from Aries Systems Corporation

Main manuscript
Click here to access/download;Manuscript;msfile-1.pdf
Click here to view linked References

A field guide for the compositional analysis of any-omics data

Thomas P. Quinn[1,2,*], Ionas Erb[3], Greg Gloor[4], Cedric Notredame[3], Mark F. Richardson[1,5,6], and Tamsyn M. Crowley[7]

[1]Bioinformatics Core Research Group, Deakin University, 3220, Geelong, Australia
[2]Centre for Molecular and Medical Research, Deakin University, 3220, Geelong, Australia
[3]Centre for Genomic Regulation (CRG), The Barcelona Institute of Science and Technology, Dr Aiguader 88, 08003, Barcelona, Spain
[4]Department of Biochemistry, University of Western Ontario, London, Ontario, Canada
[5]Genomics Centre, School of Life and Environmental Sciences, Deakin University, 3220, Geelong, Australia
[6]Centre for Integrative Ecology, School of Life and Environmental Sciences, Deakin University, 3220, Geelong, Australia
[7]Poultry Hub Australia, University of New England, 2351, Armidale, Australia
* *contacttomquinn@gmail.com*

Abstract

Next-generation sequencing (NGS) has made it possible to determine the sequence and relative abundance of all nucleotides in a biological or environmental sample. Today, NGS is routinely used to understand many important topics in biology from human disease to microorganism diversity. A cornerstone of NGS is the quantification of RNA or DNA presence as counts. However, these counts are not counts *per se*: the magnitude of the counts are determined arbitrarily by the sequencing depth, not by the input material. Consequently, counts must undergo normalization prior to use. Conventional normalization methods require a set of assumptions: they assume that the majority of features are unchanged, and that all environments under study have the same carrying capacity for nucleotide synthesis. These assumptions are often untestable and may not hold when comparing heterogeneous samples (e.g., samples collected across distinct cancers or tissues). Instead, methods developed within the field of compositional data analysis offer a general solution that is assumption-free and valid for all data. In this manuscript, we synthesize the extant literature to provide a concise guide on how to apply compositional data analysis to NGS count data. In doing so, we review zero replacement, differential abundance analysis, and within-group and between-group coordination analysis. We then discuss how this pipeline can accommodate complex study design, facilitate the analysis of vertically and horizontally integrated data, including multi-omics data, and further extend to single-cell sequencing data. In highlighting the limitations of total library size, effective library size, and spike-in normalizations, we propose the log-ratio transformation as a general solution to answer the question, "Relative to some important activity of the cell, what is changing?". Taken together, this manuscript establishes the first fully comprehensive analysis protocol that is suitable for any and all *-omics* data.

Introduction

The advent of next generation sequencing (NGS) has allowed scientists to probe biological systems in unprecedented ways. For an ever decreasing sum of money, it is possible to determine the sequence and relative abundance of all nucleotide fragments in a sample [43]. NGS works by sequencing a population of DNA fragments, including reverse transcribed RNA isolates. In addition to its general use for variant discovery and genome assembly, NGS is used to quantify relative abundances of (a) RNA species from tissue (RNA-Seq) [43], (b) organism diversity from the environment (meta-genomics) [68], (c) RNA species from the environment (meta-transcriptomics) [6], and (d) regions of the genome targeted by a protein (ChIP-Seq) [46], among others. Recently, improvements in the sequencing protocols have allowed for these measurements to be carried out at the single-cell level, with single-cell RNA-Seq being the most mature technology. All applications

share an analogous procedure whereby DNA or RNA are isolated from samples, optionally filtered by size or other property [27], converted to a cDNA library of nucleotide fragments, sequenced on a sequencer, and then aligned to a reference to quantify relative abundance. Since all data derive from the same assay, one might expect that they would undergo the same analysis. However, this is not true: rather, methods tailored for one mode of data do not generalize to another (e.g., RNA-Seq methods have inflated false discovery rates (FDR) when applied to meta-genomics data [59, 26]).

Fernandes et al. posited that the analysis of all NGS data can be conceptually unified by recognizing the compositional nature of these data [17]. By "compositional", we mean that the abundance of any one nucleotide fragment is only interpretable relative to another. This property emerges from the sequencer itself; the sequencer, by design, can only sequence a fixed number of nucleotide fragments. Consequently, the final number of fragments sequenced is constrained to an arbitrary limit so that doubling the input material does not double the total number of counts. This constraint also means that an increase in the presence of any one nucleotide fragment necessarily decreases the observed abundance of all other transcripts [8], and applies to bulk and single-cell sequencing data alike. It is especially problematic when comparing cells that produce more total RNA than their comparator (e.g., high c-Myc cells which up-regulate 90% of all transcripts without commensurate down-regulation [35]). However, even if a sequencer could directly sequence every RNA molecule within a cell, the cells themselves are compositional because of the volume and energy constraints that limit RNA synthesis, as evidenced by the observation that smaller cells of a single type contain proportionally less total mRNA [44].

Compositional data only carry relative information. Consequently, they exist in a Simplex space with one fewer dimensions than components. Analyzing relative data as if they were absolute can yield erroneous results for several common techniques [2, 21, 51] (also demonstrated in the Supplementary Information). First, statistical models which assume independence between features are flawed because of the mutual dependency between components [65]. Second, distances between samples are misleading and erratically sensitive to the arbitrary inclusion or exclusion of components [3]. Third, components can appear definitively correlated even when they are statistically independent [48]. For these reasons, compositional data pose specific challenges to the differential expression, clustering, and correlation analyses routinely applied to NGS data, as well as other data that measure the relative abundance of small molecules (e.g., spectrometric peak data [18]). For compositional NGS data, each sample is called a "composition" and each nucleotide species is called a "component" [21, 51].

There are three approaches to analyzing compositional data. First, the *normalization-dependent* approach seeks to normalize the data in order to reclaim absolute abundances. However, normalizations depend on assumptions that may not hold true outside of tightly controlled experiments. For example, popular RNA-Seq normalization methods assume that most transcripts have the same absolute abundance across samples [55, 5], an assumption that does not hold for the high c-Myc cells discussed above [35]. Second, the *transformation-dependent* approach transforms the data with regard to a reference to make statistical inferences relative to the chosen reference [2]. Third, the *transformation-independent* approach performs calculations directly on the components [42] or component ratios [25].

The latter two approaches constitute compositional data analysis (CoDA). Unlike normalization-based methods, CoDA methods will generalize to all data, relative or absolute. In this article, we describe a unified pipeline for the analysis of NGS count data, with all parts fully capable of modeling the uncertainty of lowly abundant counts. First, we show how existing CoDA software tools can be used to draw compositionally valid and biologically meaningful conclusions. Second, we illustrate how these methods can accommodate complex study design, facilitate the analysis of horizontally integrated multi-omics data, and accommodate machine learning applications. Third, we show how compositionality can systematically bias results if ignored. Finally, we conclude with a discussion of key problems associated with spike-in normalization, and show how the CoDA framework applies specifically to single-cell sequencing data.

Overview of pipeline

Pipeline scope

Our pipeline uses software tools made freely available for the R programming language. It begins with an unnormalized "count matrix" generated from the alignment and read-mapping of a sequence library. Details regarding quality control, assembly, alignment, and read-mapping are beyond the scope of this article, and have been covered extensively elsewhere (e.g., [13, 19]). This count matrix records the number of times each feature (e.g., transcript or operational taxonomic unit) appears in each sample. Most software return measurements as integer counts, although some use continuous values (e.g., Salmon quasi-counts [47]) or another proportional unit (e.g., transcripts per million (TPM) [62]). For many CoDA methods, units have no importance. However, small counts carry more uncertainty than large counts, and our pipeline can model this directly. Therefore, we recommend using unadjusted "raw counts". TPM can also be used with CoDA methods, but can bias the modelling of small counts if the library size differs greatly between samples. Otherwise, the data should not undergo further normalization or standardization, and must never contain negative values. Figure 1 provides a schematic of our unified NGS pipeline.

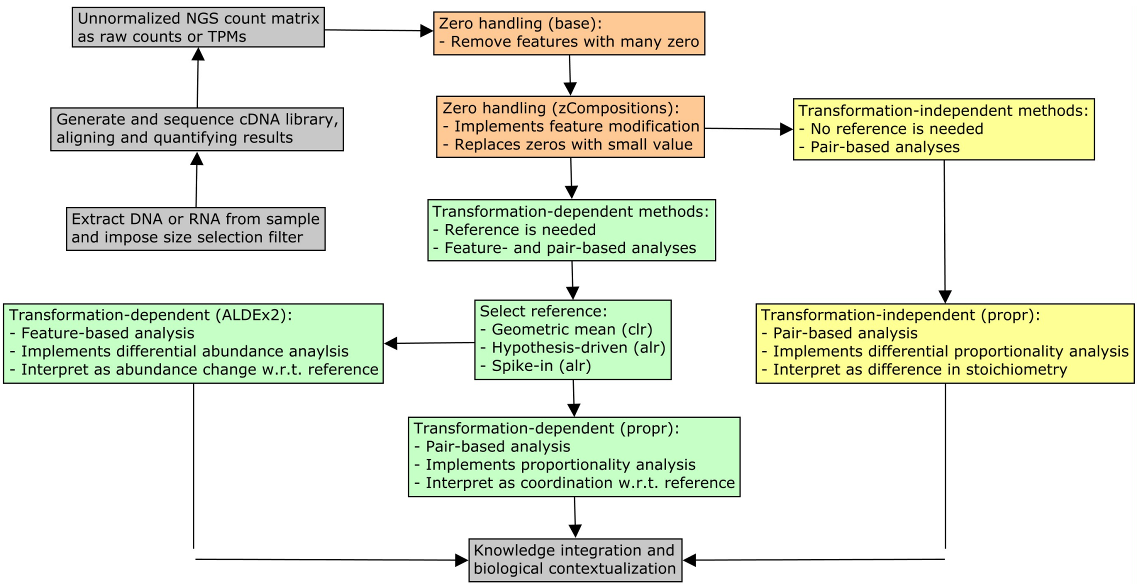

Figure 1: This figure illustrates how our unified NGS pipeline might sit within a larger workflow. Colored boxes indicate procedures that would apply to any relative data set. In orange, we describe the optional zero removal and modification steps presented in "Part 1: Zero handling". In green, we describe the log-ratio transformation-dependent methods presented in "Part 2a: Transformation-dependent analyses". This includes the differential abundance analysis of individual features and the proportionality analysis of feature pairs. In yellow, we describe the transformation-independent methods presented in "Part 2b: Transformation-independent analyses". This includes the analysis of the differences in the log-ratio means of feature pairs. In gray, we describe other essential steps unique to the data type under study but not covered here.

To demonstrate the utility of our pipeline, we use publicly available time course data of the RNA and protein expressed by mouse dendritic cells following lipopolysaccharide (LPS) exposure, a potent immunogenic stimulus. RNA-Seq and mass spectrometry (MS) data were acquired already pre-filtered to measure the relative abundance of 3147 genes in TPM-equivalent units [29]. The RNA-Seq and MS data had 28 overlapping samples, spanning 2 conditions with 7 time points and 2 replicates each.

```
rnaseq.exprs <- read.csv("rnaseq-exprs.csv")
rnaseq.annot <- read.csv("rnaseq-annot.csv")
```

Part 1: Zero handling

General strategies for zero handling

CoDA methods depend on logarithms which do not compute for zeros. Therefore, we must address zeros prior to, or during, the pipeline. Before handling zeros, the analyst must first consider the nature of the zeros. There exists three types of zeros: (1) *rounding*, also called *sampling*, where the feature exists in the sample below the detection limit, (2) *count*, where the feature exists in the sample, but counting is not exhaustive enough to see it at least once, and (3) *essential*, where the feature does not exist in the sample at all [41]. The approach to zero handling depends on the nature of the zeros [41]. For NGS data, a nucleotide fragment is either sequenced or not, and would not contain rounding zeros. Since there is no general methodology for dealing with essential zeros within a strict CoDA framework [41], we assume that any feature present in at least one sample could appear in another sample if sequenced with infinite depth, and thus treat all NGS zeros as "count zeros". Others have also suggested that the essential zeros of NGS count data are sufficiently modeled as sampling zeros [56].

There are two general approaches to zero handling. In *feature removal*, components with zeros get excluded, yielding a sub-composition that can be analyzed by any CoDA method. Feature removal is usually appropriate when a feature contains many zeros, and can always be justified for essential zeros. In *feature modification*, zeros get replaced with a non-zero value, with or without modification to non-zeros. Analysts may choose one or both zero handling procedures, but should always demonstrate that the removal or modification of zero-laden features does not change the overall interpretation of the results.

Feature modification with zCompositions

For "count zeros", Martin-Fernandez et al. recommend replacing zeros by a Bayesian-multiplicative replacement strategy that preserves the ratios between the non-zero components [41], implemented in the zCompositions package as the cmultRepl function [45]. Alternatively, one could use a multiplicative simple replacement strategy, whereby zeros get replaced with a fixed value less than 1 in a compositionally robust manner. Here, we use zCompositions to replace zeros.

```r
library(zCompositions)
# expects count matrix with rows as samples
rnaseq.no0 <- cmultRepl(rnaseq.exprs, output = "counts")
```

Part 2a: Transformation-dependent analyses

The log-ratio transformation

All components in a composition are mutually dependent features that cannot be understood in isolation. Therefore, any analysis of individual components is done with respect to a reference. This reference transforms each sample into an unbounded space where any statistical method can be used. The centered log-ratio (**clr**) transformation uses the geometric mean of the sample vector as the reference [1]. The additive log-ratio (**alr**) transformation uses a single component as the reference [1]. Other transformations use specialized references based on the geometric mean of a subset of components (collectively called multi-additive log-ratio (**malr**) transformations [50]). One **malr** transformation is the inter-quartile log-ratio (**iqlr**) transformation which uses components in the inter-quartile range of variance [69]. Importantly, transformations are not normalizations: while normalizations claim to recast the data in absolute terms, transformations do not. The results of a transformation-based analysis must be interpreted with respect to the chosen reference. Of these, the **clr** transformation is most common:

$$\text{clr}(\mathbf{x}_j) = \left[\ln \frac{x_{1,j}}{g(\mathbf{x}_j)}, ..., \ln \frac{x_{D,j}}{g(\mathbf{x}_j)}\right] \tag{1}$$

where $\mathbf{x}_j$ is the j-th sample and $g(\mathbf{x}_j)$ is its geometric mean. The other transformations replace $g(\mathbf{x}_j)$ with a different reference.

The isometric log-ratio (**ilr**) transformation uses an orthonormal basis as the reference [12], and is preferred when a non-singular covariance matrix is needed [42]. When the basis is a branch of a dendrogram, the **ilr** offers an intuitive way to contrast one set of components against another set of components. These contrasts, called balances, have been used to analyze meta-genomics data based on evolutionary trees [57, 67], but could be applied to any data if a similarly meaningful tree were available.

Differential abundance analysis with ALDEx2

Differential abundance (DA) analysis seeks to identify which features differ in abundance between experimental groups. The ALDEx2 package tests for DA in compositional data by performing univariate statistical analyses on log-ratio transformed data [16, 17]. It does so with a layer of complexity that controls for technical variation by finding the expectation of B simulated instances of the data, each sampled from the Dirichlet distribution. This procedure implicitly models the uncertainty of low counts while also handling zeros.

Importantly, ALDEx2 identifies DA *with respect to the chosen reference*. By default, this reference is the geometric mean of the composition. It is possible, if not likely, that the mean centers are not the ideal references; if so, differences in the transformed abundances would not reflect differences in the absolute abundances. On the other hand, if one could assume that the chosen reference did have fixed absolute abundance across all samples, then the log-ratio transformation can be benchmarked as a "log-ratio normalization" [51]. Under these conditions, ALDEx2 can identify DA with high precision in RNA-Seq data [17, 50], and control false positive rates in highly sparse 16S meta-genomic count data [59]. However, the "log-ratio normalization" interpretation implies a similar assumption implied by other DA tools: that the majority of transcript species remain unchanged [30]. Alternatively, one could select an arbitrary reference based on a biological hypothesis to identify *relative DA*, even if the reference does not have fixed abundance across samples. Figure 2 shows how the chosen reference changes the interpretation of DA.

To run ALDEx2, the user must provide count data with integer values, a vector of group labels, and a reference. The reference could be "all" (for **clr**), "iqlr" (for **iqlr**), or one or more user-specified features (for **alr** or **malr**). Here, we use the geometric mean of two NFκB sub-units as a hypothesis-based reference, chosen because LPS activates NFκB to control the transcription of other immune genes [49]. With this reference, up-regulation signifies that a gene's expression increases beyond that of NFκB, allowing for a clear biological interpretation. Table 1 lists 47 genes up-regulated relative to NFκB.

```
library(ALDEx2)
# expects count matrix with rows as features
ref <- grep("Nfkb", colnames(rnaseq.exprs))
tt <- aldex(t(ceiling(rnaseq.exprs)), rnaseq.annot$Treatment,
            denom = ref)
tt.bh05 <- tt[tt$we.eBH < .05,]
up <- rownames(tt.bh05[tt.bh05$effect > 0,])
```

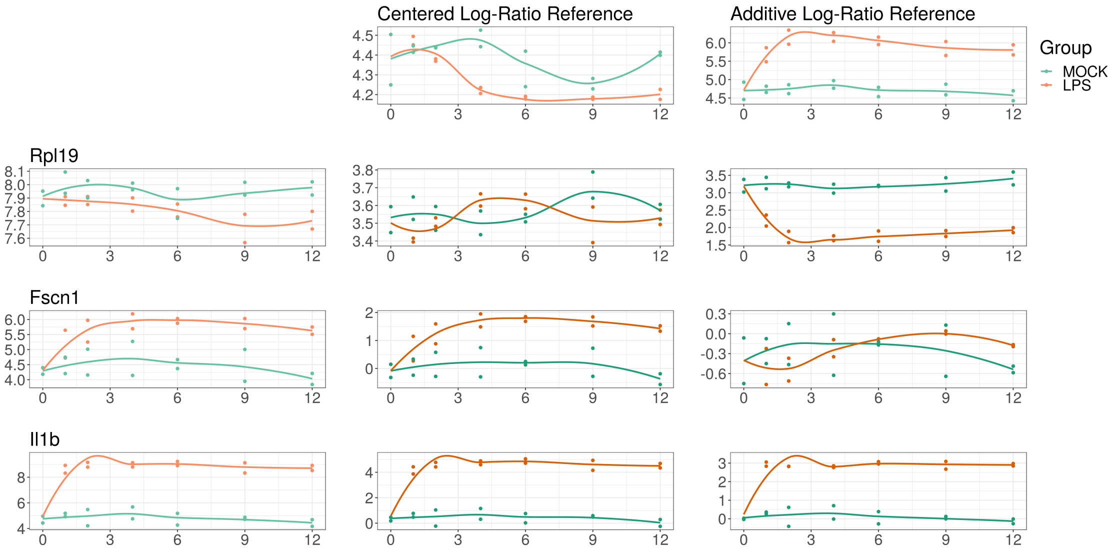

Figure 2: This figure illustrates how the interpretation of differential abundance with respect to the reference chosen. On the left margin, we show the log-abundance of three genes (RPL19, FSCN1, and IL1B) for the LPS-treated cells (orange) and control (blue). For compositional data, these abundances carry no meaning in isolation because the constrained total imposes a "closure bias". On the top margin, we show the log-abundance of two references: the geometric mean of the samples (a la the **clr**) and a hypothesis-based reference NFκB (a la the **alr**). In the middle, we show the abundance of the log-ratio of the left margin feature divided by the top margin reference (equivalent to left margin minus top margin in log space). RPL19 alone appears more abundant in the control, but actually has equivalent expression when compared with the geometric mean; however, it has significantly higher expression in the control relative to NFκB. On the other hand, FSCN1 alone appears more highly expressed in the LPS-treated cells, which remains true when compared with the geometric mean; however, it has equivalent expression relative to NFκB (interpreted as NFκB and $FSCN1$ expression changing similarly in response to LPS stimulation). IL1B alone appears more highly expressed in the LPS-treated cells, which remains true when compared with the geometric mean and with NFκB (interpreted as IL1B expression becomes even higher than NFκB expression in response to LPS stimulation). Choosing a reference makes normalization unnecessary, but requires a shift in interpretation.

Proportionality analysis with propr

Proportionality analysis is designed to identify feature coordination in compositional data [34, 14], without assuming sparsity in the association network [20, 31]. The propr package tests for the presence of feature coordination across all samples, irrespective of group label, by calculating one of three proportionality measures. The default measure, ρ_p, resembles correlation in that it ranges from $[-1, 1]$. Like DA, proportionality analysis requires a reference.

```
library(propr)
# expects count matrix with rows as samples
pr <- propr(rnaseq.no0, metric = "rho", ivar = "clr")
```

The propr package offers two alternatives to zero handling. The propr::aldex2propr function will calculate the expected proportionality from the simulated instances generated by ALDEx2, again addressing the uncertainty of low counts [7]. The alpha argument will use a Box-Cox transformation to approximate log-ratios in the presence of zeros, a pragmatic approach that allows for essential zeros, but does not fall under the strict CoDA framework [24]. For proportionality, we do not calculate parametric p-values. Instead, we permute the FDR for a given cutoff. From this, we choose the cutoff $\rho_p > 0.45$ to control FDR below 5%. The package vignette describes several built-in tools for visualizing proportionality. Figure 3 shows the output of the getNetwork function.

```
# permute FDR at rho > [0, .05, ..., .95, 1]
```

	Effect Size	Difference (between)	Difference (within)	Expected BH p-value
Il1b	4.8539	3.9487	0.6741	0.0000
Irg1	4.3102	3.8973	0.7921	0.0000
Il1a	3.7029	3.8503	0.8858	0.0000
Cd40	2.2352	5.3311	2.1057	0.0000
Ifih1	2.1541	2.9109	1.1659	0.0000
Isg15	1.9786	4.4698	1.7736	0.0000
Oasl1	1.9007	5.6498	2.0976	0.0000
Ifit1	1.7985	5.6188	2.0884	0.0000
Ptgs2	1.6242	4.1557	2.1450	0.0002
Gbp5;Gbp1	1.6166	2.4571	1.2421	0.0000
Rsad2	1.4991	6.3455	2.3854	0.0001
Marcksl1	1.4732	1.0694	0.5945	0.0001
BC006779	1.4296	2.2012	1.2946	0.0001
Mndal	1.4201	2.1379	1.5041	0.0000
Parp14	1.2784	1.7883	1.0123	0.0001
Ifit2	1.2484	5.4887	2.6462	0.0002
Clic4	1.2480	0.8639	0.5592	0.0003
Slc7a2	1.2210	1.3711	0.9898	0.0002
Ifi205	1.1996	5.3006	3.5592	0.0025
Sp140	1.1672	1.0080	0.7301	0.0005
Cmpk2	1.1063	5.7463	2.0717	0.0004
Stat5a	1.0586	0.8639	0.6706	0.0014
Ifi47	1.0162	2.0936	1.5576	0.0029
Acsl1	1.0126	1.0976	1.0462	0.0009
Pyhin1	0.9934	1.9125	1.4583	0.0025
Ifit3	0.9928	4.6974	3.2673	0.0014
Rnf213;AK217856	0.9784	1.2956	1.0600	0.0038
Daxx	0.9745	1.2166	0.8876	0.0120
IIGP;Iigp1	0.9664	3.5404	3.1808	0.0025
Ccl5	0.9601	2.0771	1.8373	0.0015
Il1rn	0.9584	0.6658	0.6290	0.0020
Irgm1	0.9390	1.6959	1.0858	0.0100
Flnb	0.9012	1.7435	1.8152	0.0100
Cd274	0.8627	0.6039	0.6514	0.0059
Trex1	0.8456	0.5742	0.5944	0.0111
Ehd1	0.7748	0.3831	0.4689	0.0084
Car13	0.7474	1.2011	1.3585	0.0121
Rasa4	0.7446	0.8603	0.9212	0.0490
Gbp3	0.7410	1.5300	1.4991	0.0131
Xaf1	0.7405	1.5211	1.3979	0.0208
Gm4902	0.7303	1.9651	1.7953	0.0155
Oas3	0.7042	1.5712	1.7536	0.0206
Serpinb2	0.7009	1.8151	2.1638	0.0242
Dhx58;D11lgp2	0.6898	1.4633	1.7083	0.0409
Gbp2	0.6696	1.5273	1.5533	0.0215
Saa3	0.5988	1.0026	1.5134	0.0185
Sbds	0.5465	0.3072	0.5514	0.0492

Table 1: This table shows the 47 genes selected as significantly up-regulated by ALDEx2 when using the NFκB sub-units as a reference. One can interpret this "up-regulation" to mean that the gene increases its expression in response to LPS stimulation more than NFκB. All p-values correspond to the expectation of the Benjamini-Hochberg adjusted p-values computed from a Welch's t-test over 128 simulated instances of the data. By choosing a reference that is relevant to the biological system under study, we can gain meaningful insights from the data without any need for normalization. In this table, between-group differences are the differences between the two conditions (defined for each Dirichlet instance), within-group differences are the maximum difference across Dirichlet instances (defined for each condition), and effect sizes are the ratio of the between-group differences to the maximum of within-group differences (defined for each Dirichlet instance). The columns "Effect size", "Difference (between)", and "Difference (within)" report the median effect size, median between-group difference, and median within-group difference, respectively.

```
pr <- updateCutoffs(pr, seq(0, 1, .05))
pr@fdr
# using a more strict cutoff
getNetwork(pr, cutoff = 0.9, col1 = up)
getResults(pr, cutoff = 0.9)
```

Proportionality depends on a log-ratio transformation and must get interpreted with respect to the chosen reference. Although proportionality appears more robust to spurious associations than correlation [34, 52], wrongly assuming that the reference has fixed absolute abundance across all samples could lead to incorrect conclusions [14]. We interpret **clr**-based proportionality to signify a coordination that follows the general trend of the data. In other words, these proportional genes move together as individuals relative to how most genes move on average.

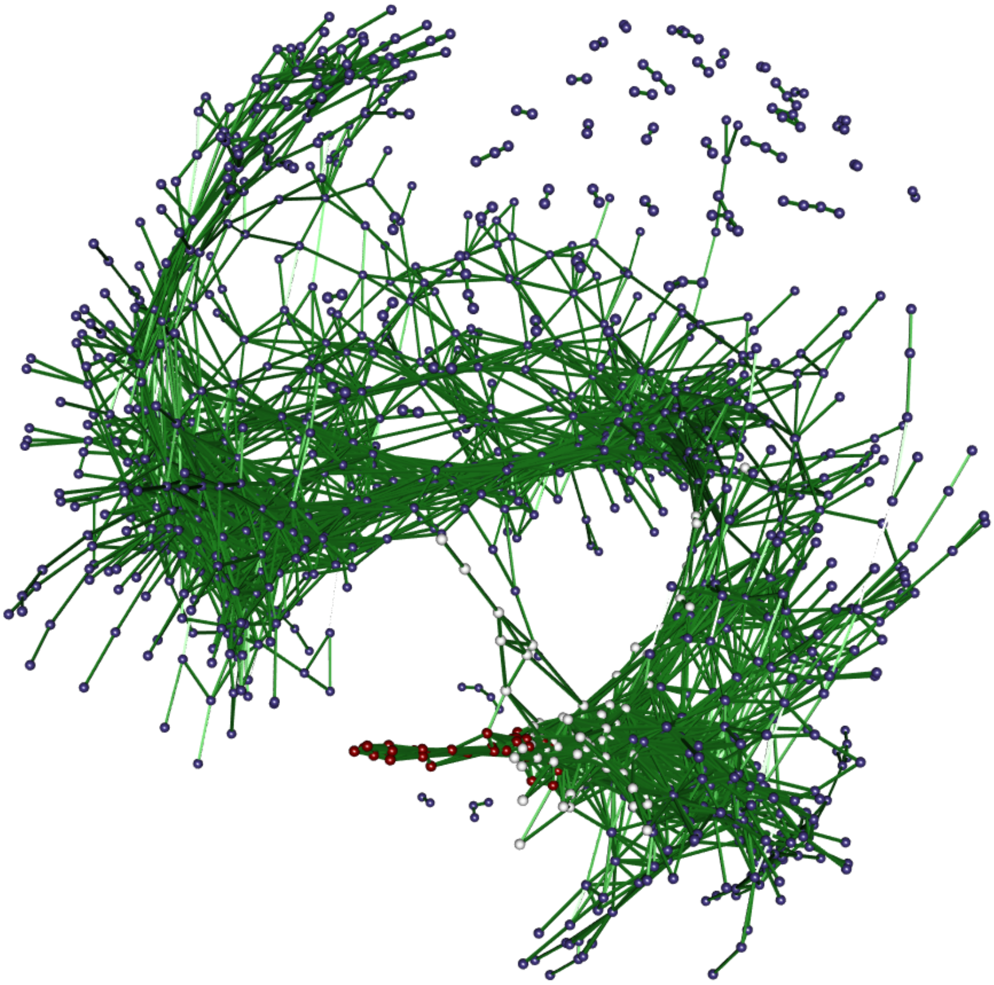

Figure 3: This figure shows a network where edges indicate a high level of coordination between gene expression relative to the per-sample geometric mean. Node color indicates differential expression relative to NFκB. The connections between red nodes indicate genes whose expression increase more than NFκB in a coordinated manner. The connections between white nodes indicate genes whose expression increase the same amount as NFκB in a coordinated manner. The connections between blue nodes indicate genes whose expression either (a) up-regulate less than NFκB, (b) do not change absolutely, or (c) down-regulate, all in a coordinated manner. The high level of connectivity between all nodes suggests a strong coordinated response to LPS. Like correlated pairs, proportional pairs can have any slope in non-log space. Note that this network only shows highly coordinated events (where $\rho_p > .9$).

Part 2b: Transformation-independent analyses

The methods above depend on a log-ratio transformation to standardize the comparison of one gene's expression (or one pair's coordination) with another. However, by comparing the variance of the log-ratios (VLR) within groups to the total VLR, we do not need a reference to estimate between-group differences in coordination [15, 66]:

$$\mathrm{VLR}^k(\mathbf{x}^g, \mathbf{x}^h) = \mathrm{var}\left[\ln \frac{x_{g,1}}{x_{h,1}}, ..., \ln \frac{x_{g,N^k}}{x_{h,N^k}}\right]. \tag{2}$$

for group k with N^k samples, where $\mathbf{x}^g$ and $\mathbf{x}^h$ are component vectors. From this equation, we see that any normalization or transformation factor would cancel. The VLR ranges from $[0, \infty)$, where zero indicates perfect coordination. Otherwise, VLR lacks a meaningful scale [1]. As such, we cannot compare the VLR of one pair to the VLR of another pair (hence why we used proportionality instead) [34, 52]. However, in differential proportionality, we compare the VLR for the same pair across groups [15].

Differential proportionality analysis is designed to identify changes in proportionality between groups [15], interpretable as a change in gene stoichiometry. The propd function tests for events where the proportionality factor (i.e., the magnitude of $\frac{x}{y}$) differs between the experimental groups. This is measured by θ_d which ranges from $[0, 1]$, where zero indicates a maximal difference between the groups. As above, users can permute the FDR and build a network, but can also calculate a parametric p-value from θ_d using the updateF function [15], with the optional application of limma::voom precision weights [32] and F-statistic moderation [58]. Precision weights eliminate the mean-variance relationship that affects the results for low counts, while the moderated statistic helps avoid false positive results in the case of few replicates. Figure 4 shows significant differentially proportional pairs containing NFκB in the log-ratio. Most of these companion genes were also called differentially abundant by ALDEx2.

```
# expects count matrix with rows as samples
pd <- propd(rnaseq.no0, group = rnaseq.annot$Treatment)
# calculate p-value
pd <- updateF(pd)
getResults(pd)
```

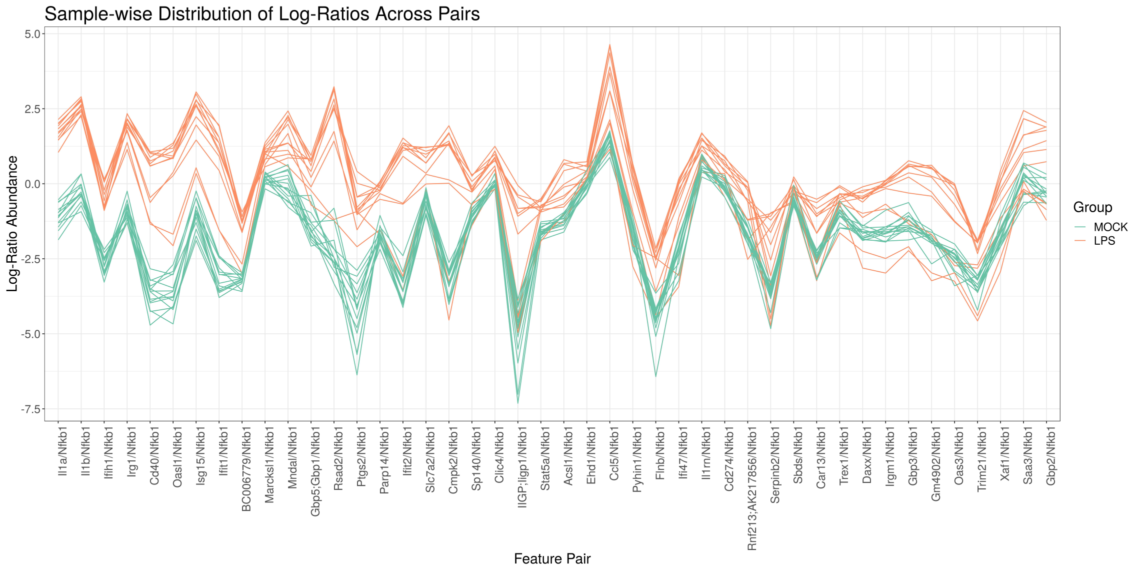

Figure 4: This figure shows a parallel coordinate plot of the log-ratio abundance (y-axis) of significant differentially proportional pairs that contain NFκB in the log-ratio (x-axis). Each line represents a single sample, colored by group. Gene pairs toward the left of the x-axis have greater differences in the log-ratio means between groups (i.e., smaller θ_d values). This plot only shows pairs for which the LPS-stimulated samples have different log-ratio means from the control (with the order of the numerator and denominator chosen such that the LPS average is always greater than the control average). It is not surprising that many of these significant pairs contain the same genes found by differential abundance analysis. Indeed, one can think of differential proportionality analysis as the differential abundance analysis of all pairwise log-ratios. Although pairs toward the right of the x-axis still have large differences in log-ratio abundance on average, some time points deviate from the trend. Indeed, this figure incidentally reveals a time-dependent process that we could test for specifically with models presented in "Complex study design".

Advanced applications

Complex study design

Above, we used our pipeline to analyze the data as if samples belonged to one of two groups. This pipeline can also accommodate complex study designs with multiple covariates. For ALDEx2, we can supply a model.matrix R object to find the expectation of a linear model (instead of a *t*-test). On the other hand, proportionality is calculated for all samples regardless of class label, and so does not require a new procedure. Differential proportionality measures the difference in the log-ratio abundance between two groups. By design, it is an efficient implementation of the two-group ANOVA expressed by the formula $[\log(\mathbf{x}_g) - \log(\mathbf{x}_h)] \sim$ group, for all combinations of features g and h. Thus, we can extend differential proportionality by modeling each pairwise log-ratio outcome as a function of any model.matrix. This may become computationally burdensome for high-dimensional data.

Vertical data integration

We envision two general strategies for the vertical integration of compositional data. First, the *row join* strategy treats other *-omics* data as additional samples and models the *-omics* source as a covariate. This requires that all *-omics* sources map to the same features. For the RNA-Seq and MS data used here, both quantify the relative abundance of gene products. This allows us to use ALDEx2 to find features where mRNA abundance changes more than protein abundance, relative to a common reference (and *vice versa*). Likewise, we can use proportionality analysis to find feature pairs where genes and proteins both have coordinated expression in response to LPS. Finally, we can use differential proportionality analysis to find feature pairs with stoichiometric differences between a gene pair and its respective protein pair. Figure 5 shows some examples of

differentially proportional pairs.

```
rna <- rnaseq.no0[rnaseq.annot$Treatment == "LPS",]
pro <- masshl.no0[masshl.annot$Treatment == "LPS",]
merge <- rbind(rna, pro)
group <- c(rep("RNA", 14), rep("Protein", 14))
pd <- propd(merge, group)
```

Second, the *column join* strategy, treats other *-omics* data as additional features. This strategy is more complicated, as it requires that each *-omics* source has its own reference. In practice, we should perform differential abundance analysis on each *-omics* source independently. For proportionality and differential proportionality analysis, we would need to log-ratio transform each *-omics* source independently, then column join them with `cbind`. Here, any proportionality occurring between features from different sources would be with respect to two references, and must get interpreted accordingly.

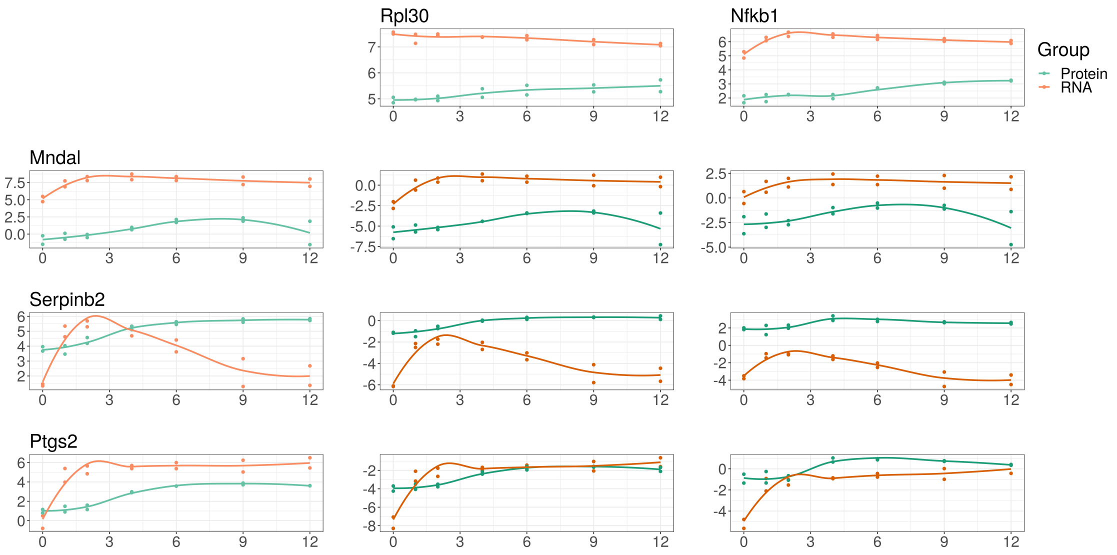

Figure 5: This figure compares mRNA abundance with newly synthesized protein abundance following LPS stimulation, illustrating the vertical integration of multi-omics data under a compositional framework. On the left margin, we show the log-abundance of three genes (MNDAL, SERPINB2, and PTGS2) as measured by RNA-Seq (orange) and mass spectrometry (blue). For compositional data, these abundances carry no meaning in isolation because the constrained total imposes a "closure bias". On the top margin, we show the log-abundance of two references: RPL30 (chosen because its abundance is proportional to the geometric mean of the samples) and NFκB (chosen based on the hypothesis). In the middle, we show the abundance of the log-ratio of the left margin feature divided by the top margin reference (equivalent to left margin minus top margin in log space). MNDAL alone appears to exist more as mRNA than protein, which remains true when compared with both references. This suggests that MNDAL is translated with lower efficiency than RPL30 and NKκB. On the other hand, SERPINB2 alone appears to exist as mRNA and protein similarly on average; however, it actually exists more as protein than mRNA when compared with both references. This suggests that $MNDAL$ is translated with greater efficiency than RPL30 and NKκB. PTGS2 alone appears to exist more as mRNA than protein, but this difference is less apparent when compared with both references. This suggests that PTGS2 is translated with a similar efficiency to RPL30 and NKκB. By choosing a reference shared between two multi-omics data sets, we can perform an analysis of vertically integrated data without any need for normalization.

Horizontal data integration

The term "mega-analysis" describes a single analysis of samples collected across multiple studies [63]. Batch effects pose a major barrier to mega-analyses. Here, we consider two types of batch effects. The first affects all genes within a sample proportionally (e.g., due to differences in sequencing depth). A log-ratio transformation will automatically remove this batch effect. The second

affects only some genes within a sample (e.g., due to differences in RNA depletion protocols). This requires explicit modification of the corrupted features. If needed, one could apply standard batch correction tools, normally applied to normalized data, to the transformed data instead (c.f., the moderated log-link `sva` in [33]).

Clustering and classification

Most distance measures lack sub-compositional dominance, meaning that it is possible to reduce the distance between samples by adding dimensions [3]. When clustering compositions, methods that rely on distance, like hierarchical clustering, also lack sub-compositional dominance [40]. Instead, one should use the Euclidean distance of **clr** transformed compositions (called the Aitchison distance) [40]. Other statistical methods used for clustering, like PCA and t-SNE, also compute distance and should also get **clr** transformed prior to analysis. When clustering components, one could use the proportionality metric ϕ_s as a dissimilarity measure [52]. This latter procedures implies a reference and must get interpreted accordingly.

How best to classify compositional data remains an open question, but **ilr** transforming the data prior to model training would grant the data favorable properties, as done for linear discriminant analysis [61]. Alternatively, one could train models on the log-ratios themselves, though this may not scale to high-dimensional data. Recently, balances have been used for feature selection and classification [54], though more work is needed to improve interpretability without compromising scalability.

Selected topics

Closure bias and the implicit reference

NGS count data measure relative abundances because of the arbitrary limit imposed by the cell, the environment, and the sequencer. This is sometimes called the "constant sum constraint" because the sum of the relative abundances must equal a constant. Anything that introduces a constant sum constraint is a kind of "closure"; all closures irreversibly make a data set relative (i.e., "closed"). One could think of a cell (in the case of RNA-Seq) or the environment (in the case of meta-genomics) as natural closures, and sequencers as technical closures.

Total library size normalizations, like TPM, are not normalizations at all: they are actually yet another closure, imposing the constant sum constraint of transcripts per million. TPMs do not convert closed sequencing data into an "open" unit such as concentration. Analyzing TPMs as if they were concentrations is theoretically flawed, and can substantially affect the modeling of cellular processes. Our own analysis indicates that in Jovanovic et al., mRNA translation rates could have been systematically over-estimated due to compositional bias. In the Supplementary Information, we show that at the latest time point, the error compared to normalized data is around 13% in the control condition, reaching 35% in LPS-stimulated samples. This bias is due to the closure operation: if the analyst does not select a reference, the estimates must get interpreted with regard to the unknown and immeasurable "closure bias". Since the magnitude of this closure bias can be large for samples that range widely in terms of nucleotide synthesis capacity, a reference should always be used when modeling the univariate features of compositional data. If a reference is not chosen, then the closure bias acts as an "implicit reference" that makes interpretation impossible.

Count compositions and low-count imprecision

Closed count data differ from idealized compositional data because additive variation affects small counts more than large counts [52]. As such, the difference between 1 and 2 counts is not the same as the difference between 1000 and 2000 counts. Moreover, NGS experiments almost always have many more features than samples, leading to severe under-estimation of the technical variance; indeed, the technical variance can be much larger than the biological variance at the low-count margin [16]. "Count zero" features are those that are observed as a non-zero value in at least one sample, and thus are expected to be observed at or near the margin in other samples. While not intuitive, the distribution of the relative "count zero" values is quite large and spans many orders of magnitude [22]. In addition, the expected value of a "count zero" feature must be greater than zero because a value greater than zero was observed in at least one sample.

As mentioned above, the "count zero" values can be modified to give a point-estimate of their expected value, but this leads to under-estimation of their true variance since we are estimating the expected value of the feature. In the approach instantiated in the aldex.clr function used by the ALDEx2::aldex.ttest, ALDEx2::aldex.effect, and propr::aldex2propr functions, a distribution of "count zero" values is determined by sampling from the Dirichlet distribution (i.e., a multivariate generalization of the β distribution). Another way to think about the Dirichlet distribution is a multivariate Poisson sampling with a constant sum constraint. The distribution of relative abundances near the low-count margin can be surprisingly wide, both as estimated by sampling from the Dirichlet distribution, and as observed in real data [22]. By sampling from the Dirichlet distribution, we get a set of multivariate probability vectors, each of which is as likely to have been observed from the underlying data as the one actually observed from the sequenced sample. From this, ALDEx2 and propr can account for low-count technical imprecision (which can be much larger than the biological variation) by reporting the expected values of a test statistic instead of the point estimate [16].

Spike-in "log-ratio normalization"

Transformations are not normalizations because they do not claim to recast the data in absolute terms. However, if one were to choose a set of references with *a priori* known fixed abundance across all samples, one could use this "ideal reference" to normalize the data (something we call a "log-ratio normalization" [51]). The use of spike-in controls, consisting of multiple synthetic nucleotide sequences with known absolute abundance, may offer one such option. For RNA-Seq, the External RNA Controls Consortium (ERCC) spike-in set consists of 92 polyadenylated RNA transcripts with varying length (250-2000 nt) and GC content (5-51%) with a 10^6-fold range in abundance [28]. The spike-in set is added to a standardized amount of purified RNA in equimolar concentrations, then both the spike-in and target transcripts are processed together to create a cDNA library. Since 23 of the ERCC transcripts are designed to have the same absolute abundance, one could use their geometric mean as a reference to recast the data in absolute terms. Similarly, one could spike-in a known quantity of bacteria cells or synthetic plasmids to standardize the abundance of PCR-amplified microbiome samples [60].

However, two important assumptions underly the use of spike-ins for normalization. First, it is assumed that the spike-in and target sequences have the same *capture efficiency of RNA conversion*, in that they are both equally affected by the technical biases of cDNA library creation. Second, it is assumed that the same amount of spike-in is added per total amount of RNA. The latter is a particular issue for bulk RNA-Seq due to the technical difficulty of adding an appropriate amount of spike-in at a cell population level [53]. However, even when controlling for technical variation, cells may produce less total RNA in one of the experimental groups [35] or over time [38]. In this case, standardizing the spike-in to the *total amount of input RNA* will invalidate this assumption. Without standardizing the spike-in to the *total number of cells*, it is impossible to reclaim absolute abundances (i.e., in units of transcripts per cell) [10], but even then could prove difficult if cells within the same batch produce varying amounts of total RNA.

Single-cell RNA sequencing

Single-cell RNA sequencing (scRNA-Seq) resembles bulk RNA-Seq, except that the RNA of individual cells are captured and barcoded separately prior to building the cDNA library [4]. This RNA capture step involves a non-exhaustive sample of the total RNA which acts as another closure operation to make the data relative. The sequencer would then re-close the already closed data. Interestingly, if the sequence libraries were then expressed in TPMs, the per-million divisor would act as yet another closure of the data. For these reasons, scRNA-Seq resembles other NGS count data in that each sample is a composition of relative parts. Like other NGS count data, it is impossible to estimate absolute RNA abundance without a per-cell spike-in reference.

scRNA-Seq analysis is described as being more difficult than bulk RNA-Seq analysis for two reasons. First, scRNA-Seq library sizes vary more between samples [37]. This is due to differences in the *capture efficiency of RNA extraction*, sequencing depth, and so-called "doublet" events where two cells get captured at once [37]. To address these differences in library size, the data are normalized by effective library size normalization or by reference normalization (via a set of house-keeping or spike-in transcripts). Effective library size normalization assumes that most genes are unchanged; this assumption is especially problematic for scRNA-Seq data because single-cell

experiments study heterogeneous cell populations [36]. Reference normalization has limitations too. House-keeping genes may not have consistent expression at the single-cell level due to transcriptional bursting or tissue heterogeneity. Meanwhile, scRNA-Seq spike-ins imply an additional assumption beyond the two assumptions for bulk RNA spike-ins: they assume that the spike-ins and endogenous transcripts are similarly affected by the *capture efficiency of RNA extraction* [36], in that they are both equally affected by the technical biases of single-cell RNA extraction. However, scRNA-Seq spike-ins appear less affected by technical factors than genes, suggesting that the stated assumptions may not hold [11]. On the other hand, instead of normalization, one could transform scRNA-Seq data with respect to any reference, so long as they interpret the results accordingly.

Second, scRNA-Seq contains many zeros. Although some zeros are described as "biological zeros" (i.e., *essential zeros*) [64], most are described as "dropout zeros". For "dropout zeros", each zero is a missing value caused by "low RNA input" in combination with "the stochastic nature of gene expression...at the single-cell level" [23]. By this definition, "dropout zeros" are simply *count zeros* caused by non-exhaustive sampling. In principle, they are no different than the zeros found in meta-genomic 16S data (which are already handled by our pipeline [16]). However, if an analyst wishes to impute zeros, rather than model them as a stochastic loss of information, there exists imputation methods designed specifically for compositional data [39, 9].

Discussion

Compositional data analysis (CoDA) provides a conceptual framework for studying relative data. In this paper, we present a collection of software tools designed for NGS count data that together form a pipeline which unifies the analysis of all compositional data, including RNA-Seq, metagenomics, single-cell and spectrometric peak data. Unlike existing pipelines, ours does not seek to normalize the data to reclaim absolute abundances. Instead, it transforms the data with regard to a reference, allowing the analyst to study any relative data set without invoking the often untestable assumptions underpinning NGS data normalization.

The CoDA framework has evolved independently from much of the alternative techniques currently applied to NGS data. Interestingly, although not explicitly tailored for compositional data, the most rigorous of the NGS methods have converged on similar solutions for handling compositional bias. They rely on effective library size normalizations (and offsets) that make use of the (pseudo-counted) log-transformed data in a manner similar to log-ratio transformations. In CoDA, such transformations are explicitly derived to address the constrained nature of the data. From this perspective, explicit references and pairwise log-ratios apply to a broader range of experiments, including less well-controlled studies where effective library size normalizations may not work. The analysis of count compositions, especially the handling of low-count imprecision, has now reached a state of maturity that allows for NGS analysis without any loss of formal rigor.

An important aspect of CoDA is that it better quantifies the coordination between features than correlation, the latter of which is often spurious when the compositional constraint is ignored. Meanwhile, applying differential abundance analysis with respect to a reference remains valid even across the most widely varying conditions. For clustering and classification, the fully ratio-based Aitchison distance provides a superior inter-sample distance that is still under-appreciated in current applications. Last but not least, CoDA opens up new perspectives with respect to the integration of big multi-omics data sets where explicit references may play an important role in the future.

1 Declarations

1.1 Ethics approval and consent to participate

Not applicable.

1.2 Consent for publication

Not applicable.

1.3 Availability of data and material

All data and scripts are publicly available at http://doi.org/10.5281/zenodo.1814338.

1.4 Competing interests

No authors have competing interests.

1.5 Funding

Not applicable.

1.6 Authors' contributions

TPQ outlined and drafted the field guide. TPQ, IE, GG, and MFR drafted the Selected Topics section. IE prepared the supplement. CN, MFR, and TMC supervised the project. All authors revised and approved the final manuscript.

1.7 Acknowledgements

Not applicable.

References

[1] J Aitchison. *The Statistical Analysis of Compositional Data*. Chapman & Hall, Ltd., London, UK, UK, 1986.

[2] J Aitchison. A concise guide to compositional data analysis. *2nd Compositional Data Analysis Workshop; Girona, Spain*, 2003.

[3] J. Aitchison, C. Barceló-Vidal, J. A. Martín-Fernández, and V. Pawlowsky-Glahn. Logratio Analysis and Compositional Distance. *Mathematical Geology*, 32(3):271–275, April 2000.

[4] Aisha A. AlJanahi, Mark Danielsen, and Cynthia E. Dunbar. An Introduction to the Analysis of Single-Cell RNA-Sequencing Data. *Molecular Therapy. Methods & Clinical Development*, 10:189–196, August 2018.

[5] Simon Anders and Wolfgang Huber. Differential expression analysis for sequence count data. *Genome Biology*, 11:R106, October 2010.

[6] Stavros Bashiardes, Gili Zilberman-Schapira, and Eran Elinav. Use of Metatranscriptomics in Microbiome Research. *Bioinformatics and Biology Insights*, 10:19–25, April 2016.

[7] Gaorui Bian, Gregory B. Gloor, Aihua Gong, Changsheng Jia, Wei Zhang, Jun Hu, Hong Zhang, Yumei Zhang, Zhenqing Zhou, Jiangao Zhang, Jeremy P. Burton, Gregor Reid, Yongliang Xiao, Qiang Zeng, Kaiping Yang, and Jiangang Li. The Gut Microbiota of Healthy Aged Chinese Is Similar to That of the Healthy Young. *mSphere*, 2(5):e00327–17, October 2017.

[8] K. Gerald van den Boogaart and Raimon Tolosana-Delgado. Descriptive Analysis of Compositional Data. In *Analyzing Compositional Data with R*, Use R!, pages 73–93. Springer, Berlin, Heidelberg, 2013.

[9] K. Gerald van den Boogaart and Raimon Tolosana-Delgado. Zeroes, Missings, and Outliers. In *Analyzing Compositional Data with R*, Use R!, pages 209–253. Springer, Berlin, Heidelberg, 2013.

[10] Kaifu Chen, Zheng Hu, Zheng Xia, Dongyu Zhao, Wei Li, and Jessica K. Tyler. The Overlooked Fact: Fundamental Need for Spike-In Control for Virtually All Genome-Wide Analyses. *Molecular and Cellular Biology*, 36(5):662–667, March 2016.

[11] Julie M. Deeke and Johann A. Gagnon-Bartsch. Stably expressed genes in single-cell RNA-sequencing. *bioRxiv*, page 475426, November 2018.

[12] J. J. Egozcue, V. Pawlowsky-Glahn, G. Mateu-Figueras, and C. Barceló-Vidal. Isometric Logratio Transformations for Compositional Data Analysis. *Mathematical Geology*, 35(3):279–300, April 2003.

[13] Pär G. Engström, Tamara Steijger, Botond Sipos, Gregory R. Grant, André Kahles, The RGASP Consortium, Gunnar Rätsch, Nick Goldman, Tim J. Hubbard, Jennifer Harrow, Roderic Guigó, and Paul Bertone. Systematic evaluation of spliced alignment programs for RNA-seq data. *Nature Methods*, 10(12):1185–1191, November 2013.

[14] Ionas Erb and Cedric Notredame. How should we measure proportionality on relative gene expression data? *Theory in Biosciences*, 135:21–36, 2016.

[15] Ionas Erb, Thomas Quinn, David Lovell, and Cedric Notredame. Differential Proportionality - A Normalization-Free Approach To Differential Gene Expression. *Proceedings of CoDaWork 2017, The 7th Compositional Data Analysis Workshop; available under bioRxiv*, page 134536, May 2017.

[16] Andrew D. Fernandes, Jean M. Macklaim, Thomas G. Linn, Gregor Reid, and Gregory B. Gloor. ANOVA-Like Differential Expression (ALDEx) Analysis for Mixed Population RNA-Seq. *PLOS ONE*, 8(7):e67019, July 2013.

[17] Andrew D. Fernandes, Jennifer Ns Reid, Jean M. Macklaim, Thomas A. McMurrough, David R. Edgell, and Gregory B. Gloor. Unifying the analysis of high-throughput sequencing datasets: characterizing RNA-seq, 16s rRNA gene sequencing and selective growth experiments by compositional data analysis. *Microbiome*, 2:15, 2014.

[18] P. Filzmoser and B. Walczak. What can go wrong at the data normalization step for identification of biomarkers? *Journal of Chromatography. A*, 1362:194–205, October 2014.

[19] Nuno A. Fonseca, Johan Rung, Alvis Brazma, and John C. Marioni. Tools for mapping high-throughput sequencing data. *Bioinformatics*, 28(24):3169–3177, December 2012.

[20] Jonathan Friedman and Eric J. Alm. Inferring correlation networks from genomic survey data. *PLoS computational biology*, 8(9):e1002687, 2012.

[21] Gregory B Gloor, Jean M Macklaim, Vera Pawlowsky-Glahn, and Juan J Egozcue. Microbiome datasets are compositional: And this is not optional. *Front Microbiol*, 8:2224, 2017.

[22] Gregory B Gloor, Jean M Macklaim, Michael Vu, and Andrew D Fernandes. Compositional uncertainty should not be ignored in high-throughput sequencing data analysis. *Austrian Journal of Statistics*, 45:73–87, September 2016.

[23] Wuming Gong, Il-Youp Kwak, Pruthvi Pota, Naoko Koyano-Nakagawa, and Daniel J. Garry. DrImpute: imputing dropout events in single cell RNA sequencing data. *BMC Bioinformatics*, 19(1):220, June 2018.

[24] Michael Greenacre. Measuring Subcompositional Incoherence. *Mathematical Geosciences*, 43(6):681–693, August 2011.

[25] Michael Greenacre. Variable Selection in Compositional Data Analysis Using Pairwise Logratios. *Mathematical Geosciences*, pages 1–34, July 2018.

[26] Stijn Hawinkel, Federico Mattiello, Luc Bijnens, and Olivier Thas. A broken promise: microbiome differential abundance methods do not control the false discovery rate. *Briefings in Bioinformatics*, August 2017.

[27] Steven R. Head, H. Kiyomi Komori, Sarah A. LaMere, Thomas Whisenant, Filip Van Nieuwerburgh, Daniel R. Salomon, and Phillip Ordoukhanian. Library construction for next-generation sequencing: Overviews and challenges. *BioTechniques*, 56(2):61–passim, February 2014.

[28] Lichun Jiang, Felix Schlesinger, Carrie A. Davis, Yu Zhang, Renhua Li, Marc Salit, Thomas R. Gingeras, and Brian Oliver. Synthetic spike-in standards for RNA-seq experiments. *Genome Research*, 21(9):1543–1551, September 2011.

[29] Marko Jovanovic, Michael S. Rooney, Philipp Mertins, Dariusz Przybylski, Nicolas Chevrier, Rahul Satija, Edwin H. Rodriguez, Alexander P. Fields, Schraga Schwartz, Raktima Raychowdhury, Maxwell R. Mumbach, Thomas Eisenhaure, Michal Rabani, Dave Gennert, Diana Lu, Toni Delorey, Jonathan S. Weissman, Steven A. Carr, Nir Hacohen, and Aviv Regev. Dynamic profiling of the protein life cycle in response to pathogens. *Science (New York, N.Y.)*, 347(6226):1259038, March 2015.

[30] M. Senthil Kumar, Eric V. Slud, Kwame Okrah, Stephanie C. Hicks, Sridhar Hannenhalli, and Héctor Corrada Bravo. Analysis and correction of compositional bias in sparse sequencing count data. *BMC Genomics*, 19(1):799, November 2018.

[31] Zachary D. Kurtz, Christian L. Müller, Emily R. Miraldi, Dan R. Littman, Martin J. Blaser, and Richard A. Bonneau. Sparse and Compositionally Robust Inference of Microbial Ecological Networks. *PLOS Computational Biology*, 11(5):e1004226, May 2015.

[32] Charity W. Law, Yunshun Chen, Wei Shi, and Gordon K. Smyth. voom: precision weights unlock linear model analysis tools for RNA-seq read counts. *Genome Biology*, 15:R29, January 2014.

[33] Jeffrey T. Leek. svaseq: removing batch effects and other unwanted noise from sequencing data. *Nucleic Acids Research*, 42(21), December 2014.

[34] David Lovell, Vera Pawlowsky-Glahn, Juan José Egozcue, Samuel Marguerat, and Jürg Bähler. Proportionality: A Valid Alternative to Correlation for Relative Data. *PLoS Computational Biology*, 11(3), March 2015.

[35] Jakob Lovén, David A. Orlando, Alla A. Sigova, Charles Y. Lin, Peter B. Rahl, Christopher B. Burge, David L. Levens, Tong Ihn Lee, and Richard A. Young. Revisiting Global Gene Expression Analysis. *Cell*, 151(3):476–482, October 2012.

[36] Aaron T. L. Lun, Fernando J. Calero-Nieto, Liora Haim-Vilmovsky, Berthold Göttgens, and John C. Marioni. Assessing the reliability of spike-in normalization for analyses of single-cell RNA sequencing data. *Genome Research*, October 2017.

[37] Aaron T.L. Lun, Davis J. McCarthy, and John C. Marioni. A step-by-step workflow for low-level analysis of single-cell RNA-seq data with Bioconductor. *F1000Research*, 5:2122, October 2016.

[38] Samuel Marguerat, Alexander Schmidt, Sandra Codlin, Wei Chen, Ruedi Aebersold, and Jürg Bähler. Quantitative analysis of fission yeast transcriptomes and proteomes in proliferating and quiescent cells. *Cell*, 151(3):671–683, October 2012.

[39] J. A. Martín-Fernández, C. Barceló-Vidal, and V. Pawlowsky-Glahn. Dealing with Zeros and Missing Values in Compositional Data Sets Using Nonparametric Imputation. *Mathematical Geology*, 35(3):253–278, April 2003.

[40] JA Martín-Fernández, C Barceló-Vidal, V Pawlowsky-Glahn, A Buccianti, G Nardi, and R Potenza. Measures of difference for compositional data and hierarchical clustering methods. In *Proceedings of IAMG*, volume 98, pages 526–531, 1998.

[41] Josep Antoni Martín-Fernández, Javier Palarea-Albaladejo, and Ricardo Antonio Olea. Dealing with Zeros. In *Compositional Data Analysis*, pages 43–58. Wiley-Blackwell, 2011.

[42] Glòria Mateu-Figueras, Vera Pawlowsky-Glahn, and Juan José Egozcue. The Principle of Working on Coordinates. In Vera Pawlowsky-Glahn and Antonella Buccianti, editors, *Compositional Data Analysis*, pages 29–42. John Wiley & Sons, Ltd, 2011.

[43] Michael L. Metzker. Sequencing technologies — the next generation. *Nature Reviews Genetics*, 11(1):31–46, January 2010.

[44] Olivia Padovan-Merhar, Gautham P. Nair, Andrew G. Biaesch, Andreas Mayer, Steven Scarfone, Shawn W. Foley, Angela R. Wu, L. Stirling Churchman, Abhyudai Singh, and Arjun Raj. Single mammalian cells compensate for differences in cellular volume and DNA copy number through independent global transcriptional mechanisms. *Molecular Cell*, 58(2):339–352, April 2015.

[45] Javier Palarea Albaladejo, Martín Fernández, and Josep Antoni. zCompositions - R package for multivariate imputation of left-censored data under a compositional approach. April 2015.

[46] Peter J. Park. ChIP-Seq: advantages and challenges of a maturing technology. *Nature reviews. Genetics*, 10(10):669–680, October 2009.

[47] Rob Patro, Geet Duggal, Michael I Love, Rafael A Irizarry, and Carl Kingsford. Salmon: fast and bias-aware quantification of transcript expression using dual-phase inference. *Nature methods*, 14(4):417, 2017.

[48] Karl Pearson. Mathematical Contributions to the Theory of Evolution. III. Regression, Heredity, and Panmixia. *Philosophical Transactions of the Royal Society of London. Series A, Containing Papers of a Mathematical or Physical Character*, 187:253–318, 1896.

[49] Eva M Pålsson-McDermott and Luke A J O'Neill. Signal transduction by the lipopolysaccharide receptor, Toll-like receptor-4. *Immunology*, 113(2):153–162, October 2004.

[50] Thomas P. Quinn, Tamsyn M. Crowley, and Mark F. Richardson. Benchmarking differential expression analysis tools for RNA-Seq: normalization-based vs. log-ratio transformation-based methods. *BMC Bioinformatics*, 19:274, July 2018.

[51] Thomas P. Quinn, Ionas Erb, Mark F. Richardson, Tamsyn M. Crowley, and Jonathan Wren. Understanding sequencing data as compositions: an outlook and review. *Bioinformatics*, 34(16):2870–2878, August 2018.

[52] Thomas P. Quinn, Mark F. Richardson, David Lovell, and Tamsyn M. Crowley. propr: An R-package for Identifying Proportionally Abundant Features Using Compositional Data Analysis. *Scientific Reports*, 7(1):16252, November 2017.

[53] Davide Risso, John Ngai, Terence P Speed, and Sandrine Dudoit. Normalization of RNA-seq data using factor analysis of control genes or samples. *Nature Biotechnology*, 32:896, aug 2014.

[54] J. Rivera-Pinto, J. J. Egozcue, V. Pawlowsky-Glahn, R. Paredes, M. Noguera-Julian, and M. L. Calle. Balances: a New Perspective for Microbiome Analysis. *mSystems*, 3(4):e00053–18, August 2018.

[55] Mark D. Robinson and Alicia Oshlack. A scaling normalization method for differential expression analysis of RNA-seq data. *Genome Biology*, 11:R25, 2010.

[56] Justin D. Silverman, Kimberly Roche, Sayan Mukherjee, and Lawrence A. David. Naught all zeros in sequence count data are the same. *bioRxiv*, page 477794, November 2018.

[57] Justin D. Silverman, Alex D. Washburne, Sayan Mukherjee, and Lawrence A. David. A phylogenetic transform enhances analysis of compositional microbiota data. *eLife*, 6, 2017.

[58] Gordon K. Smyth. Linear models and empirical bayes methods for assessing differential expression in microarray experiments. *Statistical Applications in Genetics and Molecular Biology*, 3:Article3, 2004.

[59] Jonathan Thorsen, Asker Brejnrod, Martin Mortensen, Morten A. Rasmussen, Jakob Stokholm, Waleed Abu Al-Soud, Søren Sørensen, Hans Bisgaard, and Johannes Waage. Large-scale benchmarking reveals false discoveries and count transformation sensitivity in 16s rRNA gene amplicon data analysis methods used in microbiome studies. *Microbiome*, 4:62, 2016.

[60] Andrzej Tkacz, Marion Hortala, and Philip S. Poole. Absolute quantitation of microbiota abundance in environmental samples. *Microbiome*, 6, June 2018.

[61] Raimon Tolosana Delgado. Uses and misuses of compositional data in sedimentology. *Sedimentary geology*, 280(S.I):60–79, December 2012.

[62] Cole Trapnell, Brian A. Williams, Geo Pertea, Ali Mortazavi, Gordon Kwan, Marijke J. van Baren, Steven L. Salzberg, Barbara J. Wold, and Lior Pachter. Transcript assembly and quantification by RNA-Seq reveals unannotated transcripts and isoform switching during cell differentiation. *Nature Biotechnology*, 28(5):511–515, May 2010.

[63] George C. Tseng, Debashis Ghosh, and Eleanor Feingold. Comprehensive literature review and statistical considerations for microarray meta-analysis. *Nucleic Acids Research*, 40(9):3785–3799, May 2012.

[64] Koen Van den Berge, Fanny Perraudeau, Charlotte Soneson, Michael I. Love, Davide Risso, Jean-Philippe Vert, Mark D. Robinson, Sandrine Dudoit, and Lieven Clement. Observation weights unlock bulk RNA-seq tools for zero inflation and single-cell applications. *Genome Biology*, 19(1):24, February 2018.

[65] K. Gerald van den Boogaart and R. Tolosana-Delgado. "compositions": A unified R package to analyze compositional data. *Computers & Geosciences*, 34(4):320–338, April 2008.

[66] Jan Walach, Peter Filzmoser, Karel Hron, Beata Walczak, and Lukáš Najdekr. Robust biomarker identification in a two-class problem based on pairwise log-ratios. *Chemometrics and Intelligent Laboratory Systems*, 171:277–285, December 2017.

[67] Alex D. Washburne, Justin D. Silverman, Jonathan W. Leff, Dominic J. Bennett, John L. Darcy, Sayan Mukherjee, Noah Fierer, and Lawrence A. David. Phylogenetic factorization of compositional data yields lineage-level associations in microbiome datasets. *PeerJ*, 5:e2969, 2017.

[68] John C. Wooley, Adam Godzik, and Iddo Friedberg. A Primer on Metagenomics. *PLOS Computational Biology*, 6(2):e1000667, February 2010.

[69] Jia R. Wu, Jean M. Macklaim, Briana L. Genge, and Gregory B. Gloor. Finding the centre: corrections for asymmetry in high-throughput sequencing datasets. *arXiv:1704.01841 [q-bio]*, April 2017. arXiv: 1704.01841.

Supplementary information

Click here to access/download
Supplementary Material
supp-1.pdf
