# Supplementary material for: A field guide for the compositional analysis of any-omics data
Source: Gigascience. 2019 Sep 23;8(9):giz107. doi: 10.1093/gigascience/giz107 (PMC6755255; doi:10.1093/gigascience/giz107)
Supplement: giz107_Supplementary_Analysis_S2 [file giz107_supplementary_analysis_s2.pdf]

An evaluation of zero-handling

There are many zero-handling procedures available. In this supplement, we briefly evaluate how several zero-handling procedures impact proportionality and differential proportionality analyses. We do this in 3 stages:

First, we take the LPS-treated RNA-Seq data and randomly select 500 genes that have no zeros. 500 is chosen to speed up the run-time. Early testing suggeted that this does not alter the global topology of the results.

Second, we artificially introduce zeros into the data. For each sample, we randomly turn 1%, 5%, 10%, 25%, or 50% of the values into a zero. The zero-introduction step is weighted so that the less abundant genes are more likely to become zero (i.e., the probability of turning 0 is 1 minus the proportion of that gene's abundance). After zero-introduction, we re-scale all counts so that the total library size is unchanged. Since the data are TPMs, this acts as a re-closure of the data.

Third, proportionality and differential proportionality are calculated on the zero-introduced data using one of the following zero-handling methods: a $+1$ offset, the "CZM" method from zCompositions, the aldex2propr method, the Box-Cox transformation with $\alpha = [.01, .05, .1, .5, 1]$, or a median imputation.

Here, median imputation is defined as replacing the zero genes with the median relative abundance of the non-zero genes. In a compositional framework, this causes the non-imputed genes to decrease in relative abundance and also changes the geometric mean center; however, it has no effect on the remaining non-imputed pairwise log-ratios. Note that the aldex2propr method is only available for proportionality, not differential proportionality, because the latter does not require a prior log-ratio transformation.

Zero-handling and propr

Figure 1 plots the **actual proportionality coefficient** (computed in the absence of zeros) against the **synthetic proportionality coefficient** (computed in the presence of artificial zeros). The x-facet describes what percent of genes were made zero, while the y-facet describes the zero-handling method used. Each point is a gene pair, and this point is colored by how many samples in that pair were made zero. Here, large values on the y-axis are more "significant".

With 1-5% zeros, we see that the relationship between the actual proportionality and synthetic proportionality is mostly conserved for genes that have no zeros. For pairs that contain some zeros, many are "penalized" (in the sense that the synthetic proportionality coefficient tends toward zero). However, it is concerning that some of the zero-laden pairs do yield spurious results (i.e., the synthetic proportionality equals 1 when the actual proportionality equals 0). A Box-Cox transformation with $\alpha = .5$, or a median imputation, seem like good choices because they are most robust to these spurious events.

With 25-50% zeros, every pair has at least one zero. Here, the synthetic proportionality coefficient tells us very little about the actual proportionality coefficient. Although this is not suprising when we consider that we arbitrarily eliminated one half of the total information, it is useful to know that the synthetic proportionality advantageously tends toward smaller numbers in this case (thereby reducing the false discovery rate).

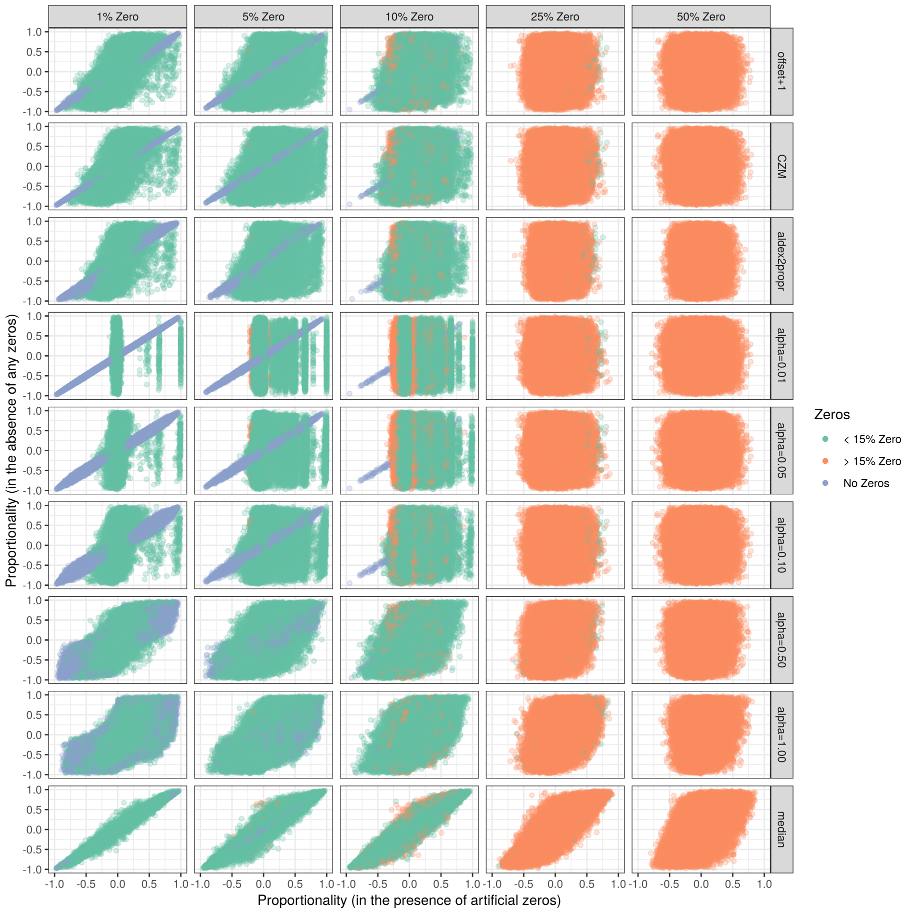

Zero-handling and propd

Figure 2 is similar to Figure 1, except that it plots the **actual differential proportionality coefficient** (computed in the absence of zeros) against the **synthetic differential proportionality coefficient** (computed in the presence of artificial zeros). Here, small values on the y-axis are more "significant".

Compared with proportionality, differential proportionality seems less prone to false positive discoveries. Instead, pairs with zeros tend to get called non-differentiated. This seems to hold true regardless of how many zeros are present.

In all cases, a Box-Cox transformation with small values heavily penalizes zero-laden pairs. Although this decreases the false positive rate, it may increase the false negative rate. On the other hand, a Box-Cox transformation with a large value, or a median imputation, seem like good choices because they provide more power to detect real differences between the zero-laden pairs without dramatically increasing the false discovery rate.

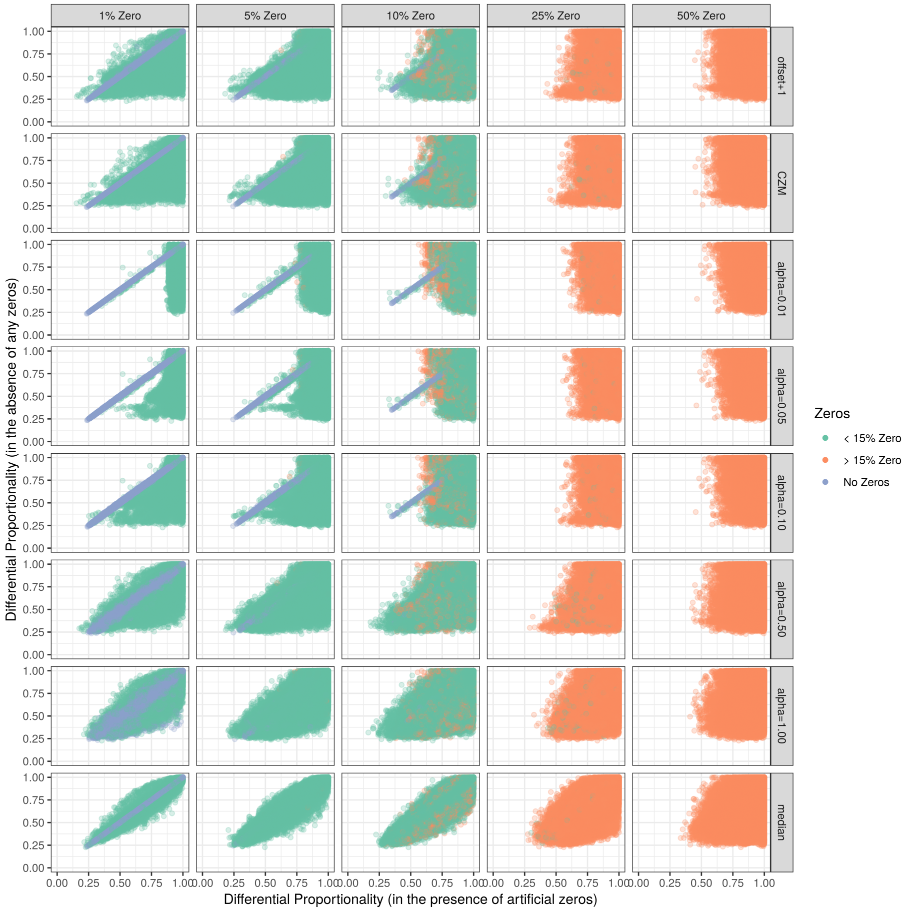

Limitations and Recommendations

We designed this benchmark to see how well the popular zero-handling procedures cope with the introduction of arbitrary zeros. While this provides us some important insights into zero-handling, it does not necessarily tell how these methods will perform for "real data" with "real zeros".

Still, it is worth considering the patterns that do emerge. First, the naive +1 offset, commonly used in bioinformatics, is not apparently worse than some sophisticated zero-handling procedures. Second, a Box-Cox transformation with small values consistently penalizes zero-laden genes, while a Box-Cox transformation with large values is more forgiving. Using a Box-Cox transformation with $\alpha = .5$ works nicely for propr and propd, and has been recommended previously [1]. Third, median imputation most closely approximates the actual proportionality and the actual differential proportionality measurements.

Why does median imputation work so well? This may have to do with how the benchmark is designed: our gold standard implies that the observed zeros are actually non-zeros. If median imputation turns the zeros back into non-zero values that are close to the original values, then it would work nicely.

Another interpretation is that most zero-handling methods treat zeros as "a small number". As such, log-ratios containing zeros will become very large, allowing a single zero to strongly impact the log-ratio variance estimate. On the other hand, median imputation implicitly treats the zeros as a "missing value" by making them the same as the "average" non-zero value. In this way, the zeros have less of an impact on the log-ratio variance estimate, allowing the non-zeros to have more influence on the final metric. Whatever the reason, the good performance of median imputation should challenge analysts to ask themselves: Do my zeros represent very small things that should be considered?, or Do my zeros represent irrelevant things that should be ignored?

More work is needed to determine whether any one zero-handling strategy is superior for all cases. We note that a Box-Cox transformation with $\alpha = .5$ does appear to improve the accuracy for propr and propd analyses, and has been recommended previously [1]. However, we still recommend that analysts try multiple zero-handling proecdures and critically evaluate the results.

References

[1] Michael Greenacre. Power transformations in correspondence analysis. *Computational Statistics & Data Analysis*, 53(8):3107–3116, June 2009.
