# Supplementary material for: A field guide for the compositional analysis of any-omics data
Source: Gigascience. 2019 Sep 23;8(9):giz107. doi: 10.1093/gigascience/giz107 (PMC6755255; doi:10.1093/gigascience/giz107)
Supplement: giz107_Response_to_Reviewer_Comments_Revision_1 [file giz107_response_to_reviewer_comments_revision_1.pdf]

Author's Response To Reviewer Comments

Close

We sincerely thank Reviewer #2 for carefully examining our supplemental code during both revision stages. Their initial suggestions have certainly made the code more useful for our readers. Unfortunately, I am unable to reproduce the error described in the minor revision comments. I have even re-installed the most up-to-date version of ALDEx2 (version 1.16.0) to make sure that recent updates did not cause a new error. Perhaps the reviewer is using an old version of ALDEx2?

Best regards,
Thom Quinn

Close
