# Supplementary material for: A field guide for the compositional analysis of any-omics data
Source: Gigascience. 2019 Sep 23;8(9):giz107. doi: 10.1093/gigascience/giz107 (PMC6755255; doi:10.1093/gigascience/giz107)
Supplement: giz107_Response_to_Reviewer_Comments_Revision_2 [file giz107_response_to_reviewer_comments_revision_2.pdf]

Author's Response To Reviewer Comments

Close

Revisions provided as requested by Scott.

Close
