# Supplementary material for: A field guide for the compositional analysis of any-omics data
Source: Gigascience. 2019 Sep 23;8(9):giz107. doi: 10.1093/gigascience/giz107 (PMC6755255; doi:10.1093/gigascience/giz107)
Supplement: giz107_Reviewer_1_Report_Original_Submission — Simon Hardwick, PhD -- 4/3/2019 Reviewed [file giz107_reviewer_1_report_original_submission.pdf]

Reviewer Report

Title: A field guide for the compositional analysis of any-omics data

Version: Original Submission **Date:** 4/3/2019

Reviewer name: Simon Hardwick, PhD

Reviewer Comments to Author:

Quinn et al. present a field guide, and accompanying software package, for the compositional analysis of high-throughput sequencing data. The framework can be applied to a wide range of -omics data, including RNA, metagenome and single-cell sequencing. Thus, it has the potential to act as an invaluable guide to researchers investigating a broad range of biological phenomena.

Major issues:

Where are the "Methods" & "Results" sections? The whole paper reads like a review article and it is unclear from the text exactly what the authors have done here (as opposed to providing a summary of existing work in the field). Were any new experiments done? If so, the details should be provided. How was the software developed?

It is noted that the authors published a review on a very similar topic last year (https://academic.oup.com/bioinformatics/article/34/16/2870/4956011); at first glance, it would seem that there is substantial overlap between the two papers.

A related concern is that the authors need to compare/benchmark their new software with previously published methods. It would be interesting to see how it performs compared to existing methods, eg. DESeq, edgeR, TMM, RUVg (to name only a few).

Minor issues:

P 1, line 52 - should be "next-generation sequencing" (hyphenated) for consistency.

P 2, lines 1-3. It is stated that all NGS applications involve alignment of reads to a reference. This statement ignores alignment-free methods, eg. k-mer based quantification and variant-calling. Whilst these may be beyond the scope of the paper, this should be stated upfront.

P 6, line 21. The following sentence appears to contain a typo: "This figure illustrates how the interpretation of differential abundance with respect to the reference chosen". Please revise.

P12, line 15: "this latter procedures" - typo. Please revise.

P 12, line 54. The authors state: "Moreover, NGS experiments almost always have many more features than samples…". This statement would often not be true for single-cell RNA-seq experiments, which now analyse many thousands of cells.

P 13, line 24: if mentioning the ERCC spike-ins, the authors should also mention other, more recent, synthetic spike-in controls for NGS, eg:

Spliced synthetic RNA spike-ins for RNA-seq:

https://www.nature.com/articles/nmeth.3958

https://www.biorxiv.org/content/10.1101/080747v1.full

Synthetic DNA spike-ins for genome sequencing:

https://www.nature.com/articles/nmeth.3957

https://jmd.amjpathol.org/article/S1525-1578(16)00046-5/fulltext
Synthetic microbial spike-ins for metagenome sequencing:
https://www.nature.com/articles/s41467-018-05555-0
P 13, lines 30-32. For this sentence - "Similarly, one could spike-in a known quantity of bacteria cells or synthetic plasmids to standardize the abundance of PCR-amplified microbiome samples." - the authors should also cite the following publication:
https://microbiomejournal.biomedcentral.com/articles/10.1186/s40168-016-0175-0
P 14, lines 2-3. The authors should provide citation(s) for this sentence: "House-keeping genes may not have consistent expression at the single-cell level due to transcriptional bursting or tissue heterogeneity".
P 14, lines 3-6. The authors state: "Meanwhile, scRNA-Seq spike-ins imply an additional assumption beyond the two assumptions for bulk RNA spike-ins: they assume that the spike-ins and endogenous transcripts are similarly affected by the capture efficiency of RNA extraction [36], in that they are both equally affected by the technical biases of single-cell RNA extraction."
I'm not sure that I understand this point. Aren't RNA spike-ins added to samples after RNA extraction; if so, how can they be affected by RNA extraction?
P 14, lines 14-15. The authors state that "dropout zeros" are caused by "the stochastic nature of gene expression...at the single-cell level". I doubt that this is correct; if stochastic gene expression led to a particular cell not expressing a given gene at a certain time, I would've thought that this would be a biological zero, not a dropout zero.
Key questions:
1) Are the methods appropriate to the aims of the study, are they well described, and are necessary controls included?
As mentioned above, the paper should be structured with clear "Methods" and "Results" sections. It is unclear whether any new experiments were done to validate/benchmark their software tool (thus I can't comment on whether necessary controls were included).
2) Are the conclusions adequately supported by the data shown?
It was unclear to me whether this study involved the generation of any experimental data. At the very least, the authors need to compare/benchmark their new software with previously published methods.
3) Please indicate the quality of language in the manuscript. Does it require a heavy editing for language and clarity?
The quality of language and writing is excellent, and in my opinion requires minimal editing (with the exception of the few minor typos raised above).
4) Are you able to assess all statistics in the manuscript, including the appropriateness of statistical tests used?
The rationale for the statistical tests and methods used in the paper are sound and well-described. However, as I am not an expert in statistics, I was not able to comprehensively assess the theoretical underpinnings of all statistical tests.

Level of Interest

Please indicate how interesting you found the manuscript: Choose an item.

Quality of Written English

Please indicate the quality of language in the manuscript: Choose an item.

Declaration of Competing Interests

Please complete a declaration of competing interests, considering the following questions:

- Have you in the past five years received reimbursements, fees, funding, or salary from an organisation that may in any way gain or lose financially from the publication of this manuscript, either now or in the future?
- Do you hold any stocks or shares in an organisation that may in any way gain or lose financially from the publication of this manuscript, either now or in the future?
- Do you hold or are you currently applying for any patents relating to the content of the manuscript?
- Have you received reimbursements, fees, funding, or salary from an organization that holds or has applied for patents relating to the content of the manuscript?
- Do you have any other financial competing interests?
- Do you have any non-financial competing interests in relation to this paper?

If you can answer no to all of the above, write 'I declare that I have no competing interests' below. If your reply is yes to any, please give details below.

I declare that I have no competing interests.

I agree to the open peer review policy of the journal. I understand that my name will be included on my report to the authors and, if the manuscript is accepted for publication, my named report including any attachments I upload will be posted on the website along with the authors' responses. I agree for my report to be made available under an Open Access Creative Commons CC-BY license (http://creativecommons.org/licenses/by/4.0/). I understand that any comments which I do not wish to be included in my named report can be included as confidential comments to the editors, which will not be published.

Choose an item.

To further support our reviewers, we have joined with Publons, where you can gain additional credit to further highlight your hard work (see: https://publons.com/journal/530/gigascience). On publication of this paper, your review will be automatically added to Publons, you can then choose whether or not to claim your Publons credit. I understand this statement.

Yes Choose an item.
