# Supplementary material for: A field guide for the compositional analysis of any-omics data
Source: Gigascience. 2019 Sep 23;8(9):giz107. doi: 10.1093/gigascience/giz107 (PMC6755255; doi:10.1093/gigascience/giz107)
Supplement: giz107_Reviewer_2_Report_Original_Submission — Amanda Charbonneau -- 4/11/2019 Reviewed [file giz107_reviewer_2_report_original_submission.pdf]

Reviewer Report

Title: A field guide for the compositional analysis of any-omics data

Version: Original Submission **Date:** 4/11/2019

Reviewer name: Amanda Charbonneau

Reviewer Comments to Author:

As a self-described field guide, this manuscript suggests a versatile pipeline for the analysis of count data, and would be a useful resource for guiding researchers who are new to this type of analysis. In contrast to most popular differential expression analysis pipelines, the authors focus on compositional analysis, which is a useful addition to the standard bioinformatics toolbox.

Major comments:

Context

Omics analysis, and RNAseq in particular, account for the vast majority of current sequencing projects, and there are a number of popular analysis pipelines and R packages already that do a good job of analyzing this type of data in statistically sound ways. While this method is a useful addition to other analysis methods, the authors don't offer any reasons why this method should be chosen over any other, or even mention other methods. I don't necessarily think the authors need to show EdgeR or DESeq2 results or provide benchmarking (although that would be nice), however some discussion of what sets this pipeline apart from these other well known methods is needed.

Accessibility

The introduction, overview and selected topics, in particular are very well written and accessible by researchers with little or no experience in -omics analysis. The authors have done a great job explaining why RNAseq data is difficult to analyse appropriately and have provided examples and intuition for the reader. To make the entire field guide equally useful to a relative novice, I would like to see this level of explanation added to several of the other sections. In particular,

   - in Part 1, the explanation for how zeros can differ is great, but there is little discussion of why the reader would choose to do this before analysis or as part of the analysis

    - in Part 2a 'The log-ratio transformation', the differences between several types of transformation are well-explained, however, with the exception of ilr, there are no guidelines for why the reader would choose one over another. This information would also be useful on page 12, line 47 where it is made clear that the choice of reference determines whether the data is interpretable.

    - in instances where you show multiple lines of code, as in lines 38-44 on page 5, it would be useful to provide more comments to explain the function of the code

   - line 16, page 12 "This latter procedures implies a reference and must get interpreted accordingly." Why does that metric imply a reference, and how does that change the interpretation?

1-pipeline.R and data

The code and data provided by the authors via zenodo has several minor errors, and also doesn't produce figure 3 as shown in the text. For Figure 3, the figure isn't printed out as a jpg like the others, which is a minor concern, however the code itself (line 120) seems to produce a plot with an entirely

different topology than the one shown in the article. Other minor points:
   - Lines 31, 32 and 33 don't run as written, and need to be changed to `output = "p-counts"`
   - The code would be much more user friendly if all of the library() calls were moved to the top of the R script so the user can tell what packages need to be installed. Even better would be to provide the install code as if statements or comments in the first few lines of the script file
   - The filenames used in the code don't match the ones used in the code callouts in the text, which is confusing
   - JovanovicMsML-counts.csv (massml) is a provided datafile, and is loaded in, subset and transposed in the R code, but doesn't get used for any analysis or explained anywhere in the code or text
Pipeline
It is very unclear when and why the authors have chosen to use the zero replaced versus raw data. In both the text and R code, they begin the analysis by using zCompositions to replace zeros in the data and make the matrix 'rnaseq.no0'. However in the next step, they use the raw data as input for ALDEx2. Since ALDEx2 can do it's own zero handling and arguably does a better job, this seems like a reasonable choice, however it makes it unclear why the zCompositions step was included. In subsequent steps, the authors use propr for both the transformation dependent and independant analyses, and note that propr has a function for ingesting the ALDEx2 simulated instances and using it's superior zero handling, however in both propr steps they use the rnaseq.no0 matrix to run the analysis. There doesn't seem to be any reason to do this, or any comparison to show that the rnaseq.no0 matrix and ALDEx2 simulated instances produce similar results. Unless there are specific instances where the zCompositions zero handling gives better results, it seems like a superfluous step in the pipeline. It should either be dropped, or have explanation added.
Minor comments
- On page 13, lines 41-43 the last sentence of the paragraph is confusing. It might help to break it into two sentences, or to specify that the 'even then' is referring back to the spike-in.
- In the Complex study design section, the authors suggest "modeling each pairwise log- ratio outcome as a function of any model.matrix". Wouldn't this pose a multiple testing problem? How would you address that given that it is more difficult to devise an appropriate simulation sampling strategy on complex designs?

- If it is possible to alter the colors, Figure 3 would be much easier to read (and more color blind friendly) if the lines were black and the nodes were the only colors.

Level of Interest

Please indicate how interesting you found the manuscript: Choose an item.

Quality of Written English

Please indicate the quality of language in the manuscript: Choose an item.

Declaration of Competing Interests

Please complete a declaration of competing interests, considering the following questions:

- Have you in the past five years received reimbursements, fees, funding, or salary from an organisation that may in any way gain or lose financially from the publication of this manuscript, either now or in the future?
- Do you hold any stocks or shares in an organisation that may in any way gain or lose financially from the publication of this manuscript, either now or in the future?
- Do you hold or are you currently applying for any patents relating to the content of the manuscript?
- Have you received reimbursements, fees, funding, or salary from an organization that holds or has applied for patents relating to the content of the manuscript?
- Do you have any other financial competing interests?
- Do you have any non-financial competing interests in relation to this paper?

If you can answer no to all of the above, write 'I declare that I have no competing interests' below. If your reply is yes to any, please give details below.

I declare that I have no competing interests

I agree to the open peer review policy of the journal. I understand that my name will be included on my report to the authors and, if the manuscript is accepted for publication, my named report including any attachments I upload will be posted on the website along with the authors' responses. I agree for my report to be made available under an Open Access Creative Commons CC-BY license (http://creativecommons.org/licenses/by/4.0/). I understand that any comments which I do not wish to be included in my named report can be included as confidential comments to the editors, which will not be published.

Choose an item.

To further support our reviewers, we have joined with Publons, where you can gain additional credit to further highlight your hard work (see: https://publons.com/journal/530/gigascience). On publication of this paper, your review will be automatically added to Publons, you can then choose whether or not to claim your Publons credit. I understand this statement.

Yes Choose an item.
